# Supplementary material for: Tailoring of a visible-light-absorbing biaxial ferroelectric towards broadband self-driven photodetection
Source: Nat Commun. 2021 Jan 12;12:284. doi: 10.1038/s41467-020-20530-4 (PMC7804191; doi:10.1038/s41467-020-20530-4)
Supplement: Supplementary file 1 — Supplementary Information [file 41467_2020_20530_MOESM1_ESM.pdf]

Supplementary Information

Tailoring of a Visible-Light-Absorbing Biaxial Ferroelectric towards Broadband Self-Driven Photodetection

Shiguo Han, Maofan Li, Yi Liu, Wuqian Guo, Mao-Chun Hong, Zhihua Sun & Junhua Luo**

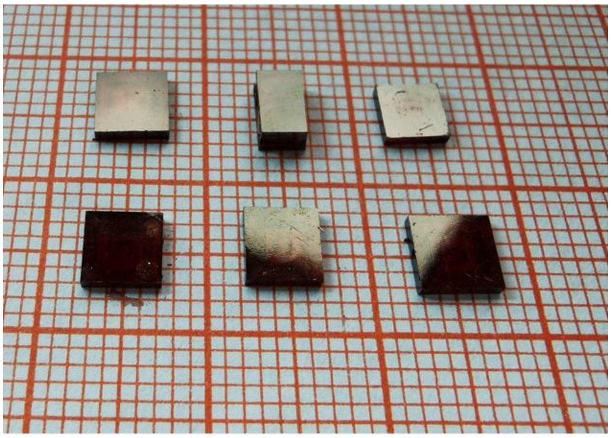

Supplementary Figure 1. Bulk crystal of PEPI grown from the concentrated HI solution by the temperature lowering method.

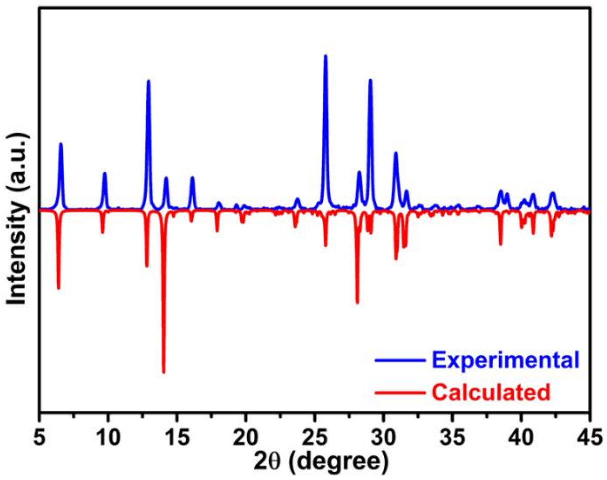

Supplementary Figure 2. Experimental and calculated PXRD patterns of PEPI at room temperature.

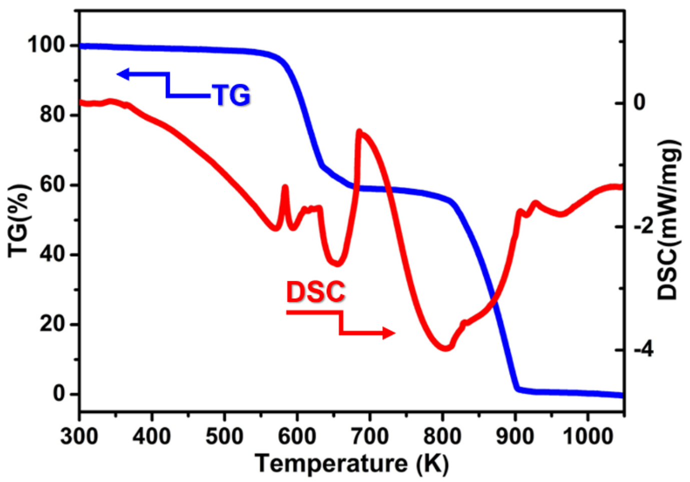

Supplementary Figure 3. The TG-DSC curves of PEPI with heating rate 10 K/min.

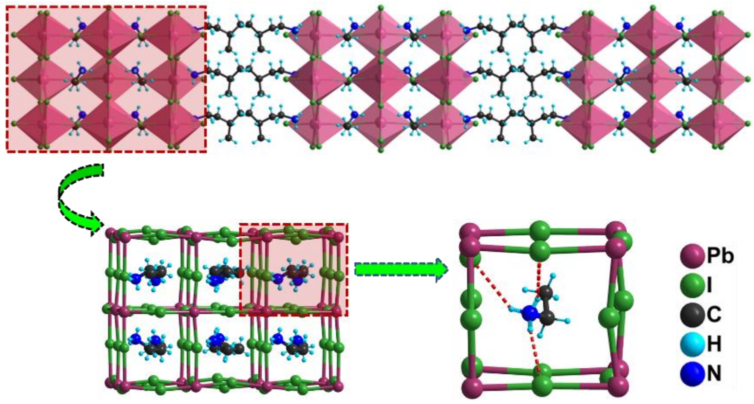

Supplementary Figure 4. The organic ethylammonium cation is confined in the cavity enclosed by distorted corner-sharing PbI_6 octahedra, being linked together by the strong N-H···I hydrogen bonds. Red imaginary lines represent the N−H···I hydrogen bonds.

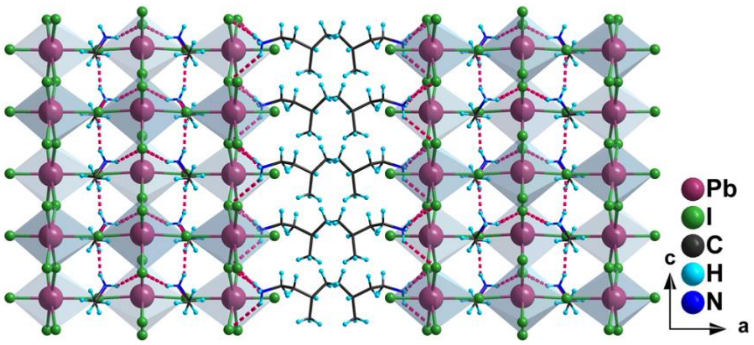

Supplementary Figure 5. Diagram of N-H···I Hydrogen bonds between inorganic perovskite sheets and organic isopentylammonium/ethylammonium cations of PEPI at FEP. Red imaginary lines represent the N−H···I hydrogen bonds.

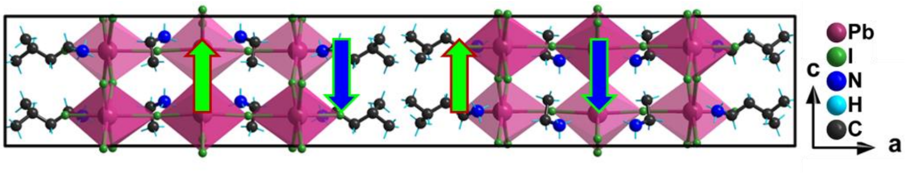

Supplementary Figure 6. The unit cell of crystal structure of PEPI at AFEP. The green and blue arrowheads denote antiparallel alignment of dipoles related to dynamic orientation of structural moieties.

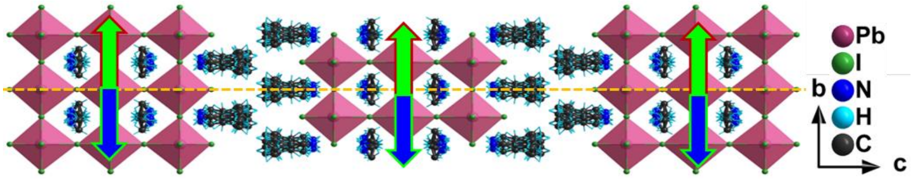

Supplementary Figure 7. Schematic representation of crystal structure for PEPI at PEP. The yellow-dashed line denotes the crystallographic mirror plane.

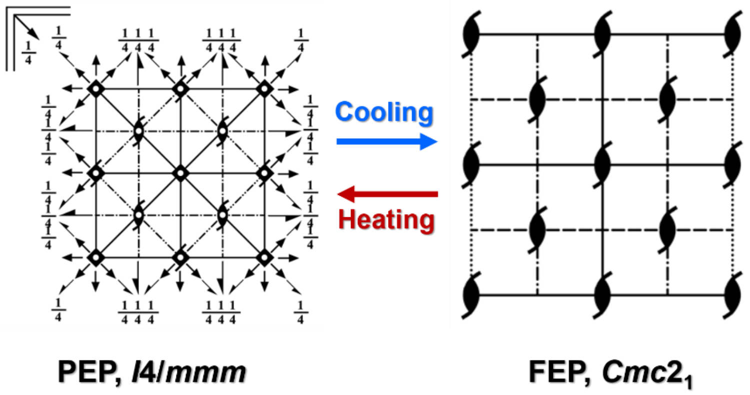

Supplementary Figure 8. Symmetry transformation occurs of PEPI from the paraelectric phase to the ferroelectric phase. The number of symmetric elements decreases by three quarters from 16 (E, $2C_4$, C_2, $2C_2'$, $2C_2''$, i, $2S_4$, σ_h, $2\sigma_v$, $2\sigma_d$) to 4 (E, C_2, σ_v, σ_v).

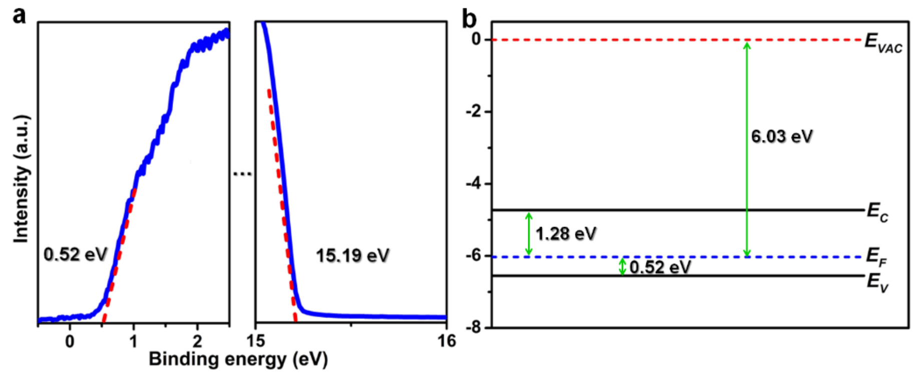

Supplementary Figure 9. (a) The ultraviolet photoelectron spectroscopy (UPS) measurement of PEPI. (b) Schematic evolution of Fermi level position and bandgap edge derived from UPS and density function theory (DFT) results. E_{VAC} is vacuum level; E_C is conduction band level; E_F is Fermi level and E_V is valence band level.

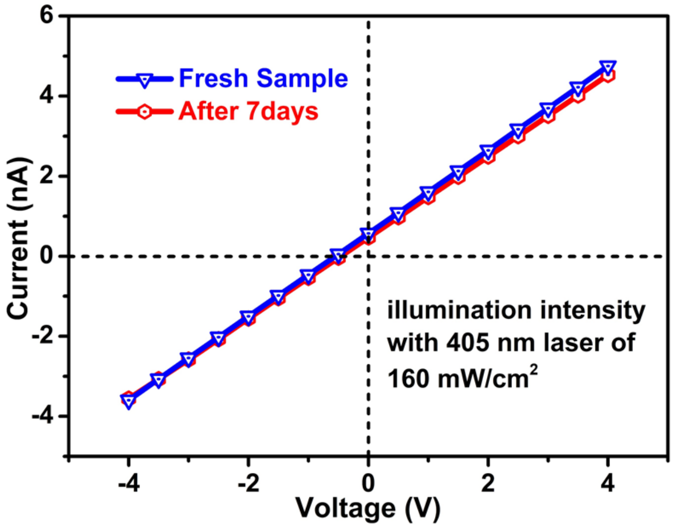

Supplementary Figure 10. Current-voltage curves of the freshly-prepared detector and the device after 7 days aging in the air under the relative humidity of 40 ± 5% at room temperature.

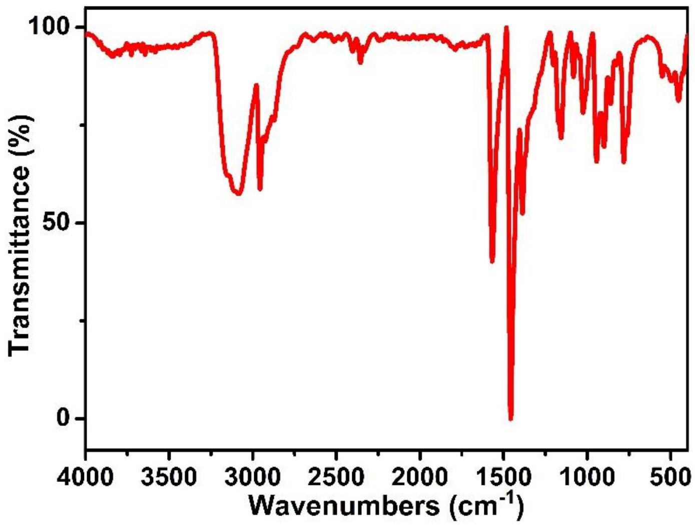

Supplementary Figure 11. Infrared spectrum for PEPI.

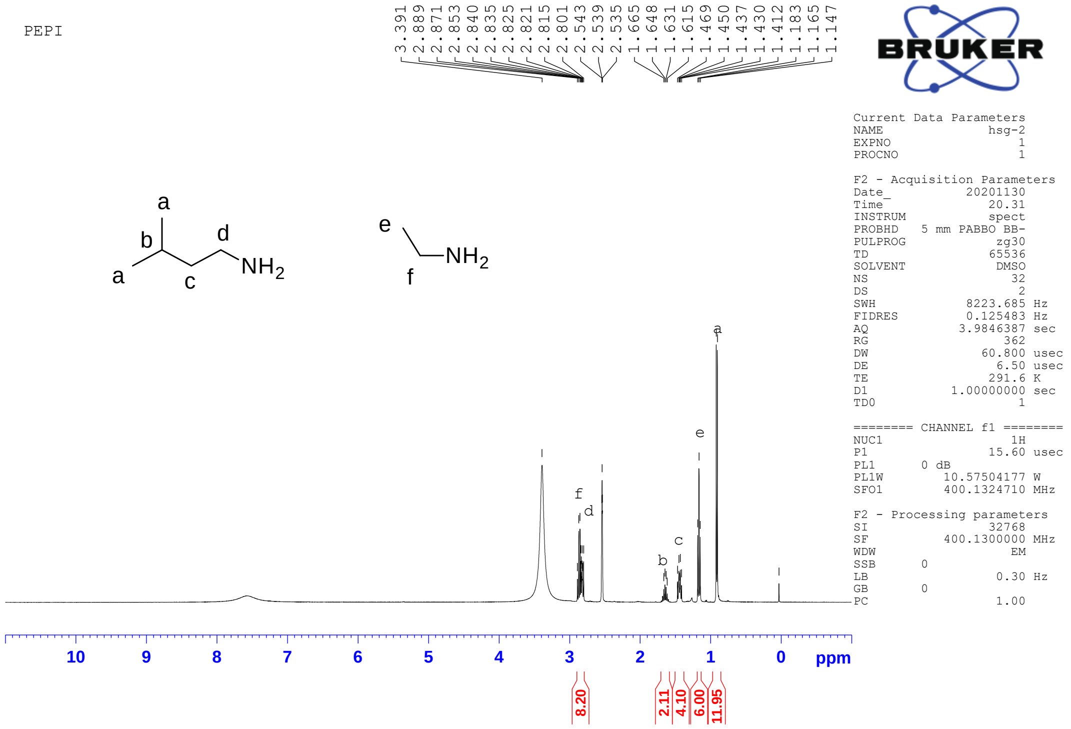

Supplementary Figure 12. [1] H-NMR of PEPI (400 MHz, DMSO-d$_6$).

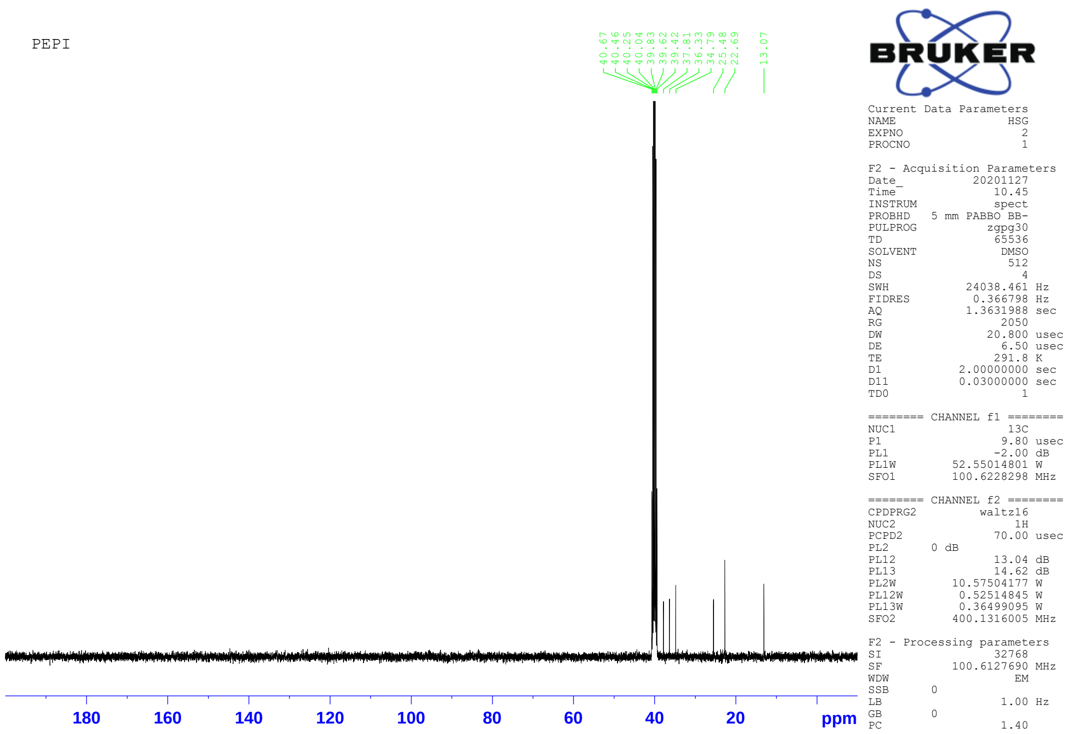

Supplementary Figure 13. ^{13}C-NMR of PEPI (400 MHz, DMSO-d_6).

Supplementary Table 1. Crystal data and structure refinement for PEPI at different temperatures: FEP (260 K); AFEP (320 K) and PEP (350 K).

Temperature	FEP (at 260 K)	AFEP (at 320 K)	PEP (at 350 K)
Empirical formula	$C_{14}H_{44}I_{10}N_4Pb_3$	$C_{14}H_{44}I_{10}N_4Pb_3$	$C_{14}H_{44}I_{10}N_4Pb_3$
Crystallographic system, space group	Orthorhombic, *Cmc*2$_1$	Orthorhombic, *Pmcn*	Tetragonal, *I4/mmm*
Cell parameters	55.225(6) Å<br>8.9515(8) Å<br>9.0019(9) Å	54.941(11) Å<br>8.9136(17) Å<br>8.9822(15) Å	6.3847(10) Å<br>6.3847(10) Å<br>55.873(9) Å
V (Å^3)	4450.1(7)	4398.8(14)	2277.6(8)
Z	4	4	2
Calculated density (g/cm^3)	3.223	3.260	3.148
F(000)	3728.0	3728.0	1864.0
Theta range (^{o})	4.426-49.996	5.082-49.998	5.834-49.97
Limiting indices	-65 ≤ h ≤ 65<br>-10 ≤ k ≤ 10<br>-9 ≤ l ≤ 10	-65 ≤ h ≤ 48<br>-10 ≤ k ≤ 9<br>-10 ≤ l ≤ 10	-7 ≤ h ≤ 7<br>-7 ≤ k ≤ 7<br>-66 ≤ l ≤ 66
Reflections collected / unique	14476/3458<br>Rint = 0.0814	17198/3878<br>Rint = 0.0964	7812/691<br>Rint = 0.0623

Data/restraints/parameters	3458/112/150	3878/113/150	691/169/100
GOF.	1.085	1.078	1.051
Final R indices [I > 2σ(I)]	R_1 = 0.0889, wR_2 = 0.2377	R_1 = 0.1301, wR_2 = 0.2768	R_1 = 0.0587 wR_2 = 0.1567

Supplementary Table 2. Bond Lengths of Pb-I of crystal PEPI at FEP (260 K).

Atom	Atom	Bond length (Å)	Atom	Atom	Bond length (Å)
Pb_2	$I_5^{\#1}$	3.256(2)	Pb_1	I_4	3.4634(17)
Pb_2	I_5	3.310(2)	Pb_1	I_3	3.1914(18)
Pb_2	$I_4^{\#2}$	3.1900(15)	Pb_1	$I_3^{\#4}$	3.1956(18)
Pb_2	I_4	3.1899(15)	Pb_1	I_1	3.182(2)
Pb_2	I_6	3.224(3)	Pb_1	$I_1^{\#5}$	3.192(2)
Pb_2	$I_6^{\#3}$	3.194(2)	Pb_1	I_2	2.993(2)

Symmetry transformations used to generate equivalent atoms:

$^{\#1}$ 1-X,1-Y,1/2+Z; $^{\#2}$ 1-X, +Y, +Z; $^{\#3}$ 1-X,2-Y,1/2+Z; $^{\#4}$ +X,1-Y, -1/2+Z; $^{\#5}$ +X, 2-Y,1/2+Z

Supplementary Table 3. Bond angles of I-Pb-I of crystal PEPI at FEP (260 K).

Bond	Bond angle (˚)	Bond	Bond angle (˚)
$I_5^{\#1}$-Pb_2-I_5	88.28(3)	I_3-Pb_1-I_4	82.09(5)
$I_5^{\#1}$-Pb_2-I_6	173.52(6)	$I_3^{\#4}$-Pb_1-I_4	82.62(5)
$I_4^{\#2}$-Pb_2-I_5	85.74(4)	I_3-Pb_1-$I_3^{\#4}$	89.61(2)
$I_4^{\#2}$-Pb_2-$I_5^{\#1}$	90.05(4)	I_3-Pb_1-I_1	178.03(6)
I_4-Pb_2-$I_5^{\#1}$	90.05(4)	I_3-Pb_1-$I_1^{\#5}$	91.46(5)
I_4-Pb_2-I_5	85.74(4)	I_1-Pb_1-I_4	96.34(6)
I_4-Pb_2-$I_4^{\#2}$	171.48(8)	$I_1^{\#5}$-Pb_1-I_4	95.85(6)
$I_4^{\#2}$-Pb_2-I_6	89.47(5)	$I_1^{\#5}$-Pb_1-$I_3^{\#4}$	178.00(6)
I_4-Pb_2-I_6	89.47(5)	I_1-Pb_1-$I_3^{\#4}$	89.01(5)
$I_6^{\#3}$-Pb_2-I_5	177.71(7)	$I_1^{\#5}$-Pb_1-I_1	89.89(2)
I_6-Pb_2-I_5	85.24(6)	I_2-Pb_1-I_4	169.50(5)
$I_6^{\#3}$-Pb_2-$I_5^{\#1}$	94.00(6)	I_2-Pb_1-$I_3^{\#4}$	90.81(7)
$I_6^{\#3}$-Pb_2-I_4	94.25(4)	I_2-Pb_1-I_3	89.70(7)
$I_6^{\#3}$-Pb_2-$I_4^{\#2}$	94.25(4)	I_2-Pb_1-$I_1^{\#5}$	90.88(7)
$I_6^{\#3}$-Pb_2-I_6	92.48(3)	I_2-Pb_1-I_1	91.72(8)

Symmetry transformations used to generate equivalent atoms:

$^{\#1}$ 1-X, 1-Y, 1/2+Z; $^{\#2}$ 1-X, +Y, +Z; $^{\#3}$ -X, 2-Y, 1/2+Z; $^{\#4}$ +X, 1-Y, -1/2+Z; $^{\#5}$ +X, 2-Y, 1/2+Z

Supplementary Table 4. N-H···I Hydrogen bonds of crystal PEPI at FEP (260 K).

D-H···A	d(D-H)	d(H···A)	< DHA	d(D···A)
N_1-H_{1C}···I_4	0.890	2.779	163.79	3.642
N_1-H_{1D}···$I_4^{\#1}$	0.890	2.865	156.97	3.700
N_1-H_{1E}···$I_6^{\#2}$	0.890	3.061	144.60	3.822
N_2-H_{2B}···I_1	0.890	2.789	131.14	3.440
N_2-H_{2C}···$I_3^{\#3}$	0.890	2.755	166.91	3.627

Symmetry transformations used to generate equivalent atoms:

$^{\#1}$ X, -Y+1, Z+1/2; $^{\#2}$ -X+1, -Y+1, Z+1/2, $^{\#3}$ X, -Y+1, Z-1/2

Supplementary Table 5. Bandgap of PEPI and some other lead-halide perovskite ferroelectric.

Compound	Bandgap	Refs
**1**	**~1.80 eV**	**This work**
$(C_4H_9NH_3)_2(C_2H_5NH_3)_2Pb_3I_{10}$	~1.90 eV	[1]
(4,4-difluorocyclohexylammnium)$_2$PbI$_4$	~2.38 eV	[2]
$[(CH_3)_3NCH_2I]PbI_3$	~2.82 eV	[3]
[4-(aminomethyl)-piperidinium]$_2$PbI$_4$	~2.38 eV	[4]
R-1-(4-chlorophenyl)-ethylammonium]$_2$PbI$_4$	~2.34 eV	[5]
S-1-(4-chlorophenyl)-ethylammonium]$_2$PbI$_4$	~2.36 eV	
$(C_4H_9NH_3)_2(CH_3NH_3)_2Pb_3Br_{10}$	~2.42 eV	[6]
$(C_4H_9NH_3)_2(CH_3NH_3)Pb_2Br_7$	~2.55 eV	[7]
$(C_4H_9NH_3)_2(NH_2CHNH_2)Pb_2Br_7$	~2.35 eV	[8]
$(C_4H_9NH_3)_2CsPb_2Br_7$	~2.68 eV	[9]
(ethylammonium)$_4$Pb$_3$Br$_{10}$	~2.70 eV	[10]
(cyclohexylammonium)$_2$PbBr$_4$	~2.95 eV	[11]
(benzylammonium)$_2$PbCl$_4$	~3.65 eV	[12]
$(C_4H_9NH_3)_2PbCl_4$	~3.49 eV	[13]

Supplementary References

[1] Han, S. G. et al. High-Temperature Antiferroelectric of Lead Iodide Hybrid Perovskites. *J. Am. Chem. Soc*. **141**, 12470-12474 (2019).

[2] Sha, T. T. et al. Fluorinated 2D Lead Iodide Perovskite Ferroelectrics. *Adv. Mater*. **31**, 1901843 (2019).

[3] Hua, X. N. et al. A Room-Temperature Hybrid Lead Iodide Perovskite Ferroelectric. *J. Am. Chem. Soc*. **140**, 12296-12302 (2018).

[4] Park, I. H. et al. Ferroelectricity and Rashba Effect in a Two-Dimensional Dion-Jacobson Hybrid Organic-Inorganic Perovskite. *J. Am. Chem. Soc.* **141**, 15972-15976 (2019).

[5] Yang, C. K. et al. The First 2D Homochiral Lead Iodide Perovskite Ferroelectrics: [R- and S-

1-(4-Chlorophenyl) ethylammonium]$_2$PbI$_4$. *Adv. Mater*. **31**, 1808088 (2019).
[6] Li, L. L. et al. Tailored Engineering of an Unusual $(C_4H_9NH_3)_2(CH_3NH_3)_2Pb_3Br_{10}$ Two‐Dimensional Multilayered Perovskite Ferroelectric for a High‐Performance Photodetector. *Angew. Chem. Int. Ed*. **56**, 12150-12154 (2017).
[7] Li, L. L. et al. Two-Dimensional Hybrid Perovskite-Type Ferroelectric for Highly Polarization-Sensitive Shortwave Photodetection. *J. Am. Chem. Soc*. **141**, 2623-2629 (2019).
[8] Li, L. L. et al. Bilayered Hybrid Perovskite Ferroelectric with Giant Two-Photon Absorption. *J. Am. Chem. Soc*. **140**, 6806-6809 (2018).
[9] Wu, Z. Y. et al. Alloying n-Butylamine into $CsPbBr_3$ To Give a Two-Dimensional Bilayered Perovskite Ferroelectric Material. *Angew. Chem. Int. Ed*. **57**, 8140-8143 (2018).
[10] Wang, S. S. et al. An Unprecedented Biaxial Trilayered Hybrid Perovskite Ferroelectric with Directionally Tunable Photovoltaic Effects. *J. Am. Chem. Soc*. **141**, 7693-7697 (2019).
[11] Sun, Z. H. et al. A Photoferroelectric Perovskite-Type Organometallic Halide with Exceptional Anisotropy of Bulk Photovoltaic Effects. *Angew. Chem. Int. Ed*. **55**, 6545-6550 (2016).
[12] Liao, W. Q. et al. A lead-halide perovskite molecular ferroelectric semiconductor. *Nature Communications* **6**, 7338 (2015).
[13] Ji, C. M. et al. The First 2D Hybrid Perovskite Ferroelectric Showing Broadband White‐Light Emission with High Color Rendering Index. *Adv. Funct. Mater.* **29**, 1805038 (2019).
